# A Hydroxypropyl Methylcellulose-Based Solid Dispersion of Curcumin with Enhanced Bioavailability and Its Hepatoprotective Activity

**DOI:** 10.3390/biom9070281

**Published:** 2019-07-15

**Authors:** Myoung-Sook Shin, Jun Sang Yu, Jaemin Lee, Young Seok Ji, Hee Joung Joung, Yu-Mee Han, Hye Hyun Yoo, Ki Sung Kang

**Affiliations:** 1 College of Korean Medicine, Gachon University, Seongnam 13120, Korea; 2 Institute of Pharmaceutical Science and Technology and College of Pharmacy, Hanyang University, Ansan 15588, Korea; 3 Dongoh Life Science Co., Ltd., #301B Venture Center, Jeonju 55069, Korea

**Keywords:** hydroxypropyl methylcellulose, curcumin, bioavailability, hepatoprotective

## Abstract

Curcumin is a polyphenol compound derived from the rhizomes of *Curcuma longa* that exhibits antioxidant, anti-inflammatory, anticancer, and antimicrobial properties. However, its low solubility in aqueous solutions, low absorption following oral administration, and rapid degradation limit its use as a functional food material. In this study, a hydroxypropyl methylcellulose-based solid dispersion of curcumin (DW-CUR 20) was prepared and its bioavailability was evaluated. In addition, its therapeutic efficacy as a hepatoprotective agent was investigated using the model of tert-butyl hydroperoxide (t-BHP)-induced hepatocyte damage. The rat plasma pharmacokinetic study showed that the oral curcumin bioavailability of DW-CUR 20 significantly increased compared to that of non-formulated curcumin. DW-CUR 20 showed a concentration-dependent hepatocyte protective effect on t-BHP-induced HepG2 cells. DW-CUR 20 inhibited the release of lactate dehydrogenase and decreased apoptosis-related proteins such as Poly (ADP-ribose) polymerase, cleaved caspase-7 and cleaved caspase-8 on t-BHP-treated HepG2 cells. These findings suggest that DW-CUR 20 could be a promising formulation for enhancing the therapeutic efficiency of curcumin and for improving the safety.

## 1. Introduction

Hydroxypropyl methylcellulose (HPMC) is a semi-synthetic dietary polymer based on cellulose. HPMC is used as a food ingredient and is employed as an emulsifier, suspending agent, film former, stabilizer, or thickener in the food industry [1]. HPMC is approved for use as a food additive by the Federal Drug Administration (FDA) and the European Medicines Agency [1]. In addition, HPMC is used as an excipient in pharmaceutical formulations to enhance the solubility of active pharmaceutical ingredients and for controlled drug delivery [2]. HPMC is generally recognized as biocompatible, biodegradable, and relatively safe [1].

Curcumin is a polyphenol compound derived from the rhizomes of *Curcuma longa* that exerts antioxidant, anti-inflammatory, anticancer, and antimicrobial activities [3,4,5]. Extensive studies have shown that curcumin plays an important role in the prevention and treatment of various chronic pro-inflammatory disorders, including liver damage [6]. Curcumin has been evaluated for its antioxidant and protective potential against liver diseases. Curcumin exerts protective effects in oxidative stress-associated liver diseases through molecular mechanisms including the suppression of lipid-peroxidation products and inflammatory cytokines. The carbon–carbon double bonds, β-diketo group, and phenyl rings of curcumin can eliminate free radicals in the cell membrane and thus it is considered a strong lipid-soluble antioxidant [7]. Curcumin can modulate the high-mobility group box 1-NF-κB translocation that prevents nonalcoholic steatohepatitis [8]. Curcumin additionally modulated the antioxidant capacity, alcohol metabolic enzyme activity, and lipid metabolism after chronic alcohol intake-induced liver injury [9]. Several reports have also identified the protective effect of curcumin on liver injury produced by drug toxicity because most anti-inflammatory, anti-analgesic, and anticancer drugs can be hepatotoxic [10]. However, its low solubility in aqueous solutions and its consequent low oral bioavailability have limited its use as a functional food material [11].

There have been several approaches related to drug formulations or drug delivery systems to improve the low oral bioavailability due to curcumin’s low solubility, and extensive studies have been conducted to develop a curcumin formulation. These studies have utilized liposomes [12], nano-emulsions [13], micelles [14], and nanostructured lipid carriers [15] to enhance the bioavailability of curcumin. However, there could be some toxicity concerns due to the surfactants and organic solvents used to prepare those formulations. In this context, the use of a solid dispersion based on HPMC could be an effective alternative to enhance the bioavailability of curcumin with minimal toxicity.

In this study, we evaluated the bioavailability of the curcumin/HPMC solid dispersion formulation and investigated its therapeutic efficacy as a hepatoprotective agent using the model of tert-butyl hydroperoxide (t-BHP)-induced hepatocyte damage.

## 2. Materials and Methods

### 2.1. Chemicals and Reagents

DW-CUR 20 was obtained from Dongoh Life Science Co., Ltd. (Jeonju, Korea). Minimum essential medium (MEM) containing Earle’s salts and L-glutamine and penicillin/streptomycin were purchased from Life Technologies (Waltham, MA, USA). Tert-butyl hydroperoxide (t-BHP), dithiothreitol, and phenylmethysulfonyl fluoride were obtained from Sigma-Aldrich (St. Louis, MO, USA). CytoTox96 Non-Radioactive Cytotoxicity Assay was purchased from Promega (Madison, WI, USA). An EZ-Cytox Enhanced Cell Viability Assay Kit was obtained from DoGen (Seoul, Korea). Fetal bovine serum (FBS) and DPBS were purchased from Welgene (Gyeongsangbuk-do, Korea). Primary antibodies against cleaved caspase-7 (D6H1) and Bcl-2 (D17C4) were purchased from Cell Signaling Technology (Denver, MA, USA). In addition, anti-β-actin (I-19) was purchased from Santa Cruz Biotechnologies (Santa Cruz, CA, USA).

### 2.2. Preparation of an Amorphous Solid Dispersion of Curcumin

An amorphous solid dispersion of curcumin (DW-CUR 20) was prepared using a facile solution mixing method with slight modifications from a previous study [16]. Briefly, hydroxypropyl methylcellulose (HPMC) was added to a final concentration of 20% in distilled water. Curcumin was dissolved in 75% ethanol and heated with constant stirring. The HPMC solution and the curcumin solution were mixed so that curcumin content was 20% of the total solid content. The solvent in the mixed solution was removed using a rotary evaporator (BÜCHI, Flawil, Switzerland). The solid dispersion powder of curcumin was obtained using a spray dryer (Labplant UK, North Yorkshire, UK).

### 2.3. Scanning Electron Microscope (SEM)

The surface morphology of curcumin and DW-CUR 20 was analyzed by a Hitachi SU5000 SEM (Tokyo, Japan). Samples were mounted on a brass stub using tape and platinum-coated for 200 s.

### 2.4. Differential Scanning Calorimetry (DSC)

DSC analysis was performed using a DSCQ20 thermal analyzer PerkinElmer DSC7 thermal analyzer (PerkinElmer, Boston, MA, USA). The DSC analysis was carried out under a nitrogen atmosphere from 30 to 250 °C at a heating rate of 10 °C/min.

### 2.5. Pharmacokinetic (PK) Experiments

Male Sprague-Dawley rats (8-week-old, weighing approximately 270–300 g) were purchased from Orient Bio. (Songnam, Korea). Rats were housed in a temperature (23 ± 2 °C) and humidity (55 ± 10%) controlled room, with a 12-h light/dark cycle, and allowed access to food and water ad libitum. One day before curcumin or DW-CUR 20 administrations, the rats were anesthetized with Zoletil^®^ (Virbac, Carros, France) and Rompun^®^ (Bayer, Leverkusen, Germany) and one femoral artery of each rat was cannulated for blood sampling using PE-50 tubing (Becton Dickinson, Lincoln Park, NJ, USA). The cannulae were fixed to the head and neck. The rats were fasted overnight with free access to water prior to curcumin or DW-CUR 20 administrations. Curcumin or DW-CUR 20 was dissolved in distilled water. Rats were divided into two groups: curcumin and DW-CUR 20. Rats were orally administered curcumin (50 mg/kg) or DW-CUR 20 (250 mg/kg), with equivalent amounts of curcumin. Aliquots of approximately 250−300 μL of heparinized blood samples were collected via a femoral artery cannula at 0 (to serve as a control), 0.083, 0.25, 0.5, 0.75, 1, 2, 4, 6, 8, 12, and 24 h after oral administration. Blood samples were centrifuged to isolate plasma, and 100 μL aliquots of plasma from each sample were stored at −20 °C until analysis. Cannulae were flushed with approximately 200 μL of heparinized 0.9% NaCl injectable solution (10 U/mL) immediately after each blood sampling to prevent occlusion. All animal procedures were approved by the Institutional Animal Care and Use Committee of Hanyang University (2018-0119A).

### 2.6. Sample Preparation for PK Experiments

Aliquots of plasma samples (0.1 mL) were transferred to 1.5-mL tubes. Acetate buffer (150 µL; pH 4.54; 0.1 M) and 5 μL of glucuronidase/arylsulfatase were added. The resulting solutions were incubated to hydrolyze curcumin at 37 °C for 1 h. Then, 1 mL of ethyl acetate with 0.2 μM terfenadine (internal standard) was added as extraction solvent. After vortexing for 1 min, samples were centrifuged at 13,200 rpm for 5 min. Supernatants (950 μL) were transferred to 1.5-mL microtubes and evaporated to dryness under a stream of nitrogen at 40 °C. The dried extracts were reconstituted in 100 μL of methanol. After vortexing for 2 min and centrifuging at 13,200 rpm for 5 min, the supernatants (5 μL) were analyzed by liquid chromatography. Calibration standards were prepared by adding appropriate concentrations (1, 3, 10, 30, 100, 300, 1000, and 3000 ng/mL) of stock solutions of curcumin (10 μL) to blank rat plasma (90 μL). Calibration standards were analyzed by liquid chromatography. The calibration curve for curcumin was linear over the investigated concentration range with a correlation coefficient (r) greater than 0.99.

### 2.7. UPLC–MS/MS Analysis

The UPLC–MS/MS system consisted of a Waters Acquity UPLC and a Waters Acquity TQD mass spectrometer with an electrospray ionization source (Waters Corporation, Milford, MA, USA). A Phenomenex Kinetex C18 column (2.1 × 50 mm, 2.6 μm) was used for chromatographic separation. Column temperature was maintained at 35 °C using a temperature-controlled column oven. The mobile phases consisted of 0.1% acetic acid in distilled water (solvent A) and 0.1% acetic acid in acetonitrile (solvent B). Isocratic elution was performed at a flow rate of 0.45 mL/min. The injection volume was 5 μL. The isocratic elution profile consisted of a 60:40 ratio of A:B for a runtime of 3.0 min. Mass detection was performed in positive ion mode. The source temperature was 150 °C, the desolvation temperature was 400 °C, and the capillary voltage was 3.0 kV. Nitrogen was used as the desolvation gas at a flow rate of 800 L/h and as the cone gas at 10 L/h. For selected reaction monitoring analyses, the precursor–product ion pairs used were *m/z* 369 → 177 for curcumin and *m/z* 472 → 436 for terfenadine.

### 2.8. PK Analysis

Pharmacokinetic parameters were determined by non-compartmental analysis using WinNonlin (Pharsight Corporation, Mountain View, CA). The total area under the plasma concentration-time curve from time zero to the last measured time (AUClast) was calculated using the trapezoidal rule method. The peak plasma concentration (C_max_) and time to reach C_max_ (T_max_) were determined directly from the experimental data.

### 2.9. Cell Culture

HepG2 cells were purchased from the Korean Cell Line Bank (KCLB) (Seoul, Korea) and maintained in Minimum Essential Media (MEM) supplemented with 10% Fetal Bovine Serum (FBS), 100 U/mL penicillin, 100 μg/mL streptomycin at 37 °C in a humidified chamber with an atmosphere of 5% CO_2_ and 95% air. The cell growth medium was changed every 2–3 days and cells were sub-cultured at 70–80% confluence. The cells were plated at appropriate cellular densities according to each experimental design.

### 2.10. Cytotoxicity Assay

The effect of DW-CUR 20 on HepG2 cell cytotoxicity was examined with EZ-Cytox Enhanced Cell Viability Assay Kit. Cells were seeded on 96-well plates (5 × 10^4^ cells/well) and treated with various concentrations of DW-CUR 20 for 12 h at 37 °C in a humidified atmosphere of 5% CO_2_ and 95% air.

### 2.11. Cytoprotective Effects

HepG2 cells were seeded in 96-well plates at a density of 5 × 10^4^ cells/well. After 24 h of incubation, the culture medium was replaced with FBS-free medium containing DW-CUR 20. After 6 h of incubation, HepG2 cells were treated with 1 mM of t-BHP for 6 h to induce cytotoxicity. The cytoprotective effect of the samples was then evaluated by EZ-Cytox Enhanced Cell Viability Assay Kit. For lactate dehydrogenase (LDH) release assay, HepG2 cells were treated with 1 mM of t-BHP for 90 min to induce secretion of LDH enzyme. LDH activity was measured according to the manufacturer’s instructions.

### 2.12. Immunoblotting Analysis

Western blot was performed as follow previous studies with some modifications [17,18]. In brief, HepG2 cells were seeded onto 6-well plates (2 × 10^4^ per well) and treated with the indicated concentrations of samples for 6 h. Whole-cell extracts were prepared using RIPA buffer supplemented with 1 mM dithiothreitol (Wako, Osaka, Japan) and protease inhibitor cocktail tablets (Roche diagnostics GmbH, Germany). Protein concentrations of each sample were adjusted to a constant amount following analysis using BCA Protein Assay Kit (Thermo Scientific, Rockford, IL, USA). Proteins were separated by electrophoresis on a 4–15% Mini-PROTEAN TGX gel (Bio-Rad, CA, USA), transferred to PVDF membranes (0.45 μm) and treated with specific primary and secondary antibodies. Antibodies were visualized and analyzed using ECL Western Blotting Detection Reagents (Thermo Scientific, Rockford, IL, USA) and Fusion Solo (Vilber Lourmat, Paris, France) following the manufacturer’s instructions.

### 2.13. Hepatoprotection in a t-BHP-Induced Hepatotoxicity Mouse Model

Male BABL/c mice were purchased from Raon Bio (Gyeonggi, Korea) at 8 weeks of age and allowed to acclimatize for 7 days in a specific pathogen-free (SPF) environment under constant conditions (temperature: 23 ± 2 °C; humidity: 50 ± 5%; light/dark cycle: 12 h) at a facility in Gachon University. All animal studies were performed according to the guidelines of the Ethics Committee for Use of Experimental Animals and approved by the Institutional Animal Care and Use Committee of Gachon University (GIACUC-R2017006-1). Mice were placed in five groups of six mice each. Animals received four successive doses of curcumin and DW-CUR 20 at the indicated doses at 0, 24, 48, 72, and 96 h, and a single dose of t-BHP (hepatotoxicity inducer) was administered intraperitoneally to mice at 73–74 h. Curcumin was used as a positive control. Control animals received only saline and the t-BHP group animals received saline instead of DW-CUR 20. Test group animals were administered curcumin (80 mg/kg) and DW-CUR 20 (200 and 400 mg/kg) orally in 0.5% methylcellulose suspensions. Blood samples were collected through the retro orbital plexus after 24 h of t-BHP administration. The samples were allowed to clot, and serum was separated by centrifugation (1200× *g*, 15 min) then stored in −80 °C for experiments. The activity of alanine aminotransferase (ALT) and aspartate aminotransferase (AST) was measured with a kit (ASAN Pharmaceutical Inc, Korea) according to the manufacturer’s instructions and previous report [19].

### 2.14. Histopathological Examination

At necropsy, the left lobe of liver was collected and was fixed in 10% buffered formalin solution. The fixed tissues were trimmed, processed, embedded in paraffin, sectioned with a microtome at 5 μm thickness and placed on glass microscope slides. Then, the sections were stained with hematoxylin and eosin (H&E) and were examined by light microscopy.

### 2.15. Statistical Analysis

Data are expressed as the mean ± standard deviation of duplicate or triplicate experiments. Statistical analysis was performed using Student’s t-test or one-way analysis of variance (ANOVA) followed by Tukey’s post hoc test. A significant difference was defined as *p* < 0.05.

## 3. Results

### 3.1. Curcumin/HPMC Solid Dispersions

**Figure 1:**
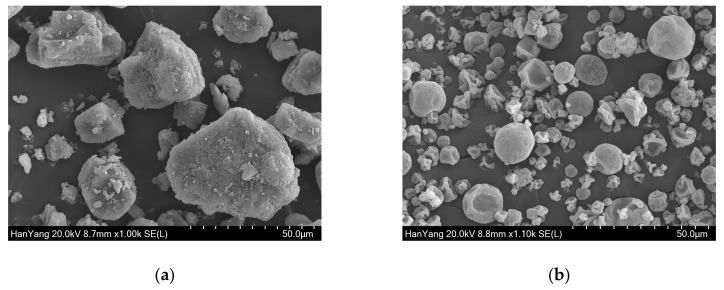
SEM images of (**a**) curcumin and (**b**) DW-CUR 20.

In this study, an HPMC-based solid dispersion of curcumin was prepared to improve the bioavailability of curcumin. The resulting solid dispersion appeared as a light yellow, dry, and fine powder. Figure 1 shows the representative SEM images for curcumin and DW-CUR 20. In the SEM images, the curcumin particles were shown to be crystalline and irregularly shaped, and the particle size varied from <10 μm to 50 μm (Figure 1a). However, DW-CUR 20 had the appearance of a typical freeze-dried substance with a regular and round shape and an even size when compared to curcumin (Figure 1b). This indicates that the morphology of the curcumin particle was clearly changed through the formation of solid dispersions with HPMC.

**Figure 2:**
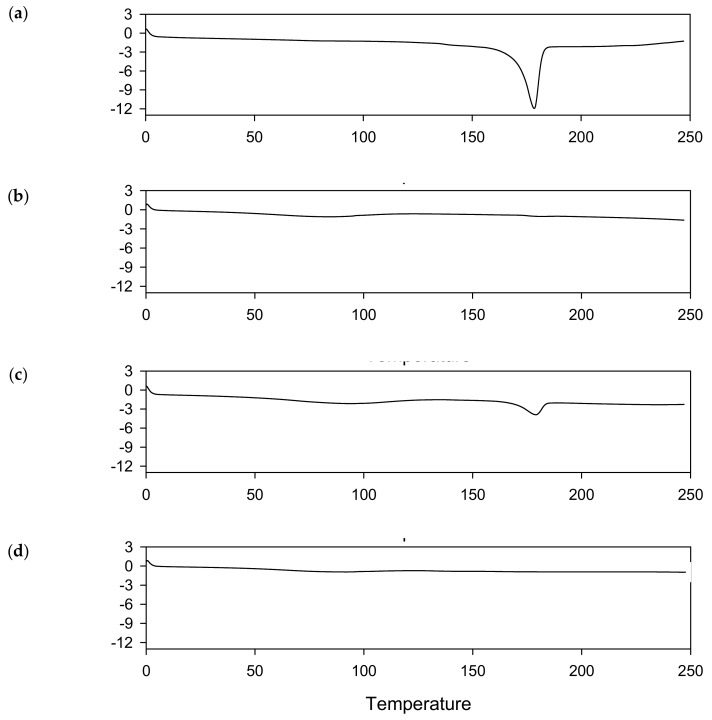
DSC thermograms of (**a**) curcumin, (**b**) HPMC, (**c**) physical mixture, and (**d**) DW-CUR 20.

DSC analyses were performed to identify whether an amorphous solid dispersion of curcumin was formed. The DSC thermograms of curcumin and DW-CUR 20 are shown in Figure 2. Curcumin and the physical mixture of curcumin and HPMC exhibited a melting endotherm at ca. 180 °C. However, DW-CUR 20 did not show any endothermic peaks in the examined temperature range. These data indicated that the curcumin in DW-CUR 20 exists in an amorphous form. The physicochemical properties of DW-CUR 20 were further characterized and confirmed based on X-ray diffraction pattern analysis and Fourier-transform infrared spectroscopy (data not shown). All these observations demonstrated that the HPMC-based amorphous solid dispersion of curcumin was successfully developed.

### 3.2. Plasma PK Studies

**Figure 3:**
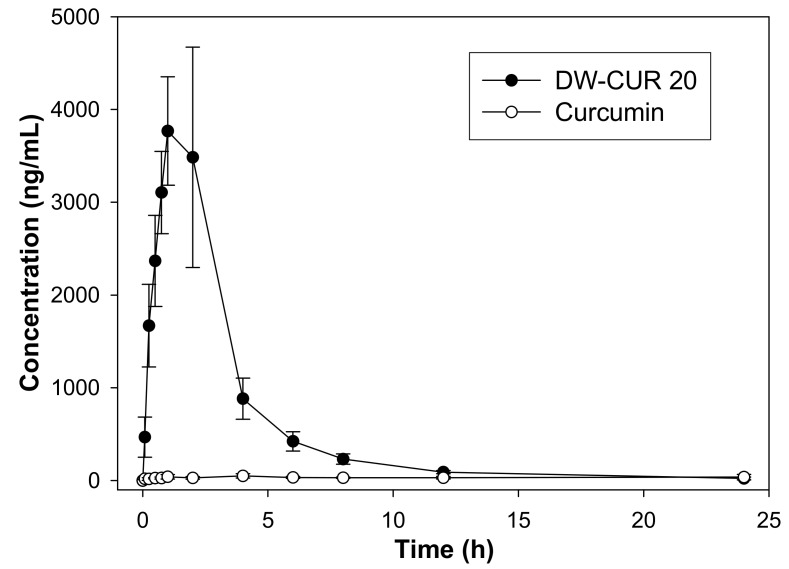
Plasma concentration–time profile of curcumin after oral administration of curcumin (50 mg/kg) and DW-CUR 20 curcumin (250 mg/kg; 50 mg/kg as curcumin) to rats (*n* = 5). Data are expressed as mean ± SD.

We conducted pharmacokinetic studies to determine whether the bioavailability of DW-CUR 20 was better than that of curcumin. The mean arterial plasma concentration-time profiles of curcumin after the oral administration of curcumin at doses of 50 mg/kg and of DW-CUR 20 at doses of 250 mg/kg (50 mg/kg as curcumin) in rats are shown in Figure 3, and the relevant pharmacokinetic parameters are listed in Table 1. The curcumin group showed generally low plasma concentrations of curcumin. The T_max_ was not clear, and the concentration variations at each time point were fairly large. These results reflected low curcumin solubility, which limited absorption in the gastrointestinal (GI) tract, resulting in the low bioavailability of curcumin. In the DW-CUR 20 group, curcumin was absorbed rapidly in the rat GI tract after oral administration. Curcumin was detected in the plasma obtained at the first blood sampling time point (5 min). Peak curcumin concentrations occurred at 1–2 h, then the plasma concentrations gradually decreased, and curcumin was detected only at low levels after the 12-h time point. The C_max_ and AUC values were 4135.0 ± 808.9 and 13,528.4 ± 3025.8, respectively. The mean AUC of the DW-CUR 20 group was more than 17 times higher than that of the curcumin group. Thus, the bioavailability of curcumin in DW-CUR 20 was at least 17 times greater than that of non-formulated curcumin.

### 3.3. Effect of DW-CUR 20 on the Viability of HepG2 Cells

**Figure 4:**
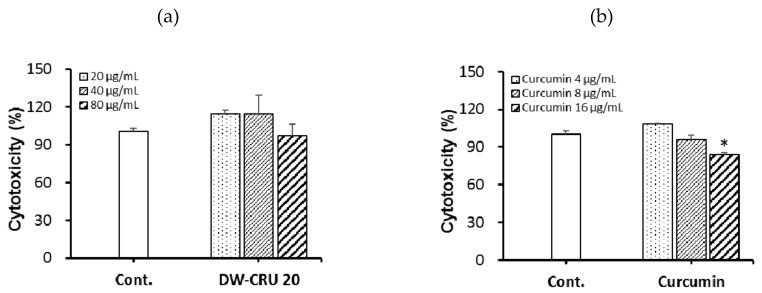
Cytotoxicity of DW-CUR 20 in HepG2 cells. Cell viability was determined using the EZ-CYTOX cell viability kit. Cells were pretreated with the indicated concentrations of (**a**) DW-CUR 20 (20, 40, and 80 μg/mL) or of (**b**) curcumin (4, 8 and 16 μg/mL) for 12 h. Data shown represent mean ± SD of triplicate experiments. * *p* < 0.05 vs. the control group.

Cytotoxicity of DW-CUR 20 was measured using an EZ-CYTOX cell viability assay kit. As shown in Figure 4, no cytotoxicity was observed across the concentration range of DW-CUR 20 (20 μg/mL to 80 μg/mL) and curcumin (4 μg/mL to 8 μg/mL). However, some morphological changes of cells were observed at 80 μg/mL of DW-CUR 20 group. Therefore, subsequent experiments were performed at concentrations that were not cytotoxic to HepG2 cells.

### 3.4. Protective Effect Against t-BHP-Induced Cytotoxicity by DW-CUR 20 in HepG2 Cells

**Figure 5:**
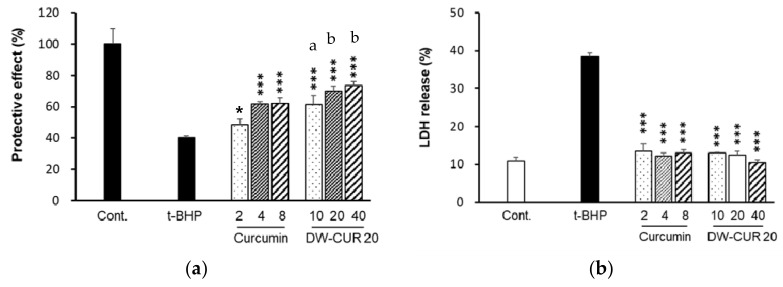
Protective effect of DW-CUR 20 against t-BHP-induced cell death in HepG2 cells. Cells were pretreated with the indicated concentrations of DW-CUR 20 (10, 20, and 40 μg/mL) or curcumin (2, 4, and 8 μg/mL) for 6 h in FBS-free medium, followed by addition of t-BHP (final concentration 1 mM). After 6 h, cells were washed twice with PBS then analyzed for cell viability using EZ-CYTOX cell viability kit (**a**). Cells were pretreated with the indicated concentrations (10, 20, and 40 μg/mL) of DW-CUR 20 for 6 h in FBS-free medium, followed by addition of t-BHP (final concentration 1000 µM). After 90 min, supernatants were collected for LDH analysis. LDH was analyzed using CytoTox96 non-radioactive cytotoxicity assay kit and calculated according to manufacturer’s protocol (**b**). Data are presented as the mean ± SD of three independent experiments. * *p* < 0.05, *** *p* < 0.001 vs. the t-BHP group. ^a^ *p* < 0.05, ^b^ *p* < 0.01 vs. the corresponding curcumin group.

T-BHP is an organic peroxide that is generally used to induce hepatotoxicity through the activation of oxidative processes [20]. Treatment of HepG2 cells with t-BHP induced apoptosis, which was reversed by the curcumin DW-CUR 20 complex. As shown in Figure 5a, the cell survival rate was about 40% when t-BHP treated at 1 mM for 6 h. However, the cell survival rate was significantly increased following treatment with curcumin or DW-CUR 20 complex. In particular, DW-CUR 20 (2, 4, and 8 µg/mL as curcumin) treatment showed greater cytoprotective activities than curcumin treatment at the corresponding concentrations (Figure 5a). These data suggest that DW-CUR 20 complex possess enhanced cytoprotective activity than pure curcumin.

Lactate dehydrogenase (LDH) is an enzyme that converts lactate to pyruvate. Under stressed conditions, LDH is released from the cell and accumulates in the extracellular space. Therefore, we evaluated the inhibition of LDH enzyme release by the curcumin DW-CUR 20 complex following t-BHP-induced cell damage in HepG2 cells. As shown in Figure 5b, following treatment with t-BHP for 90 min, the LDH release increased by approximately 40%, which was more than four times that of the control group. However, curcumin and DW-CUR 20 complex treatment significantly reduced t-BHP-induced LDH release (Figure 5b). These findings showed that DW-CUR 20 exerted cytoprotective activity (Figure 5a–b).

### 3.5. Effect of DW-CUR 20 on t-BHP-Induced Apoptosis in HepG2 Cells

**Figure 6:**
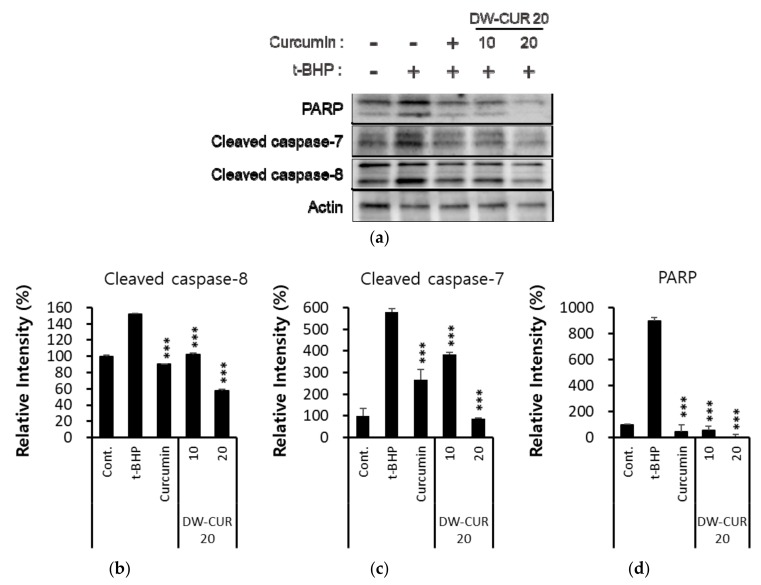
Effects of DW-CUR 20 on cleavage of caspases-7, -8 and PARP in HepG2 cells. Curcumin (4 µg/mL) was used as a positive control. Whole cell lysates were separated by electrophoresis, transferred onto membranes, and probed with the indicated antibodies. Cleaved caspase-7, -8 and PARP were detected using specific primary antibodies. β-Actin served as an internal loading control. All immunoblot bands were obtained from the same cell lysates (**a**). The bar charts display the intensity of Cleaved caspase-7, -8 or PARP after being normalized by β-Actin using ImageJ software (**b**–**d**). Data are presented as the means ± SD of three independent experiments. *** *p* < 0.001 vs the t-BHP treatment group.

Apoptosis is mainly divided into two pathways: the extrinsic apoptosis pathway, which activates the death receptor located in the cell membrane and activates caspases and poly (ADP-ribose) polymerase (PARP) to induce apoptosis [21,22,23], and the intrinsic apoptosis pathway, which mainly occurs through mitochondria and involves various proteins including Bcl-2 family proteins [24]. Caspases possess proteolytic activities that are able to cleave proteins at aspartic acid residues, and when caspases are activated, cell death is not reversed. To date, ten major caspases have been identified and categorized into initiators (caspase-2, -8, -9, -10), effectors (caspase-3, -6, -7) and inflammatory caspases (caspase-1, -4, -5) [22]. Activated caspase-8 cleaves downstream effector caspases such as caspase-3, and -7 and activates PARP, ultimately elicits the apoptosis including cell shrinkage and DNA fragmentation. Cell death induced by t-BHP was inhibited by DW-CUR 20 complex. The activation of caspase-7, -8 and PARP were observed in HepG2 cells treated with t-BHP for 6 h and caspase-7, -8 and PARP activity was inhibited by pretreatment with DW-CUR 20 (10 and 20 µg/mL). Moreover, 20 µg/mL of DW-CUR 20 (4 µg/mL as curcumin) treatment showed more increased anti-apoptotic activity than pure curcumin (4 µg/mL) (Figure 6).

### 3.6. Effect of DW-CUR 20 on t-BHP-Induced Hepatotoxicity in Mice

**Figure 7:**
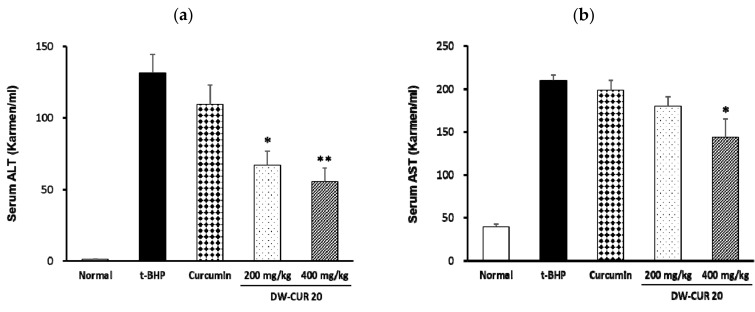
Effects of DW-CUR 20 on serum biochemical parameters. (**a**) Alanine aminotransferase (ALT) and (**b**) aspartate aminotransferase (AST) levels in t-BHP-induced hepatotoxicity in mice. Data were expressed as mean ± SE (*n* = 6). * *p* < 0.05, ** *p* < 0.01 compared to the t-BHP group.

Alanine aminotransferase (ALT/GPT) and aspartate aminotransferase (AST/GOP) are released from cells in response to hepatic tissue damage. When hepatocyte damage occurs, ALT release is increased. Hepatoprotective drugs decrease extracellular ALT. Therefore, we investigated the inhibition of hepatocytotoxicity by DW-CUR 20 using experimental animals. Curcumin (80 mg/kg) was used as a positive control, and 400 mg/kg of DW-CUR 20 contained equivalent amount of curcumin to 80 mg/kg of curcumin. As shown in Figure 7, administration of t-BHP increased the ALT and AST levels, indicating liver damage in BALB/c mice. In addition, increased ALT and AST in response to t-BHP was suppressed by DW-CUR 20 in a dose-dependent manner. Furthermore, 200 mg/kg of DW-CUR 20 significantly inhibited increased serum ALT level, and 400 mg/kg of DW-CUR 20 significantly inhibited both serum ALT and AST levels (Figure 7). However, 80 mg/kg of curcumin (corresponding to 400 mg/kg of DW-CUR 20) did not showed a statistically significant difference in the serum ALT and AST levels under the present experimental condition.

**Figure 8:**
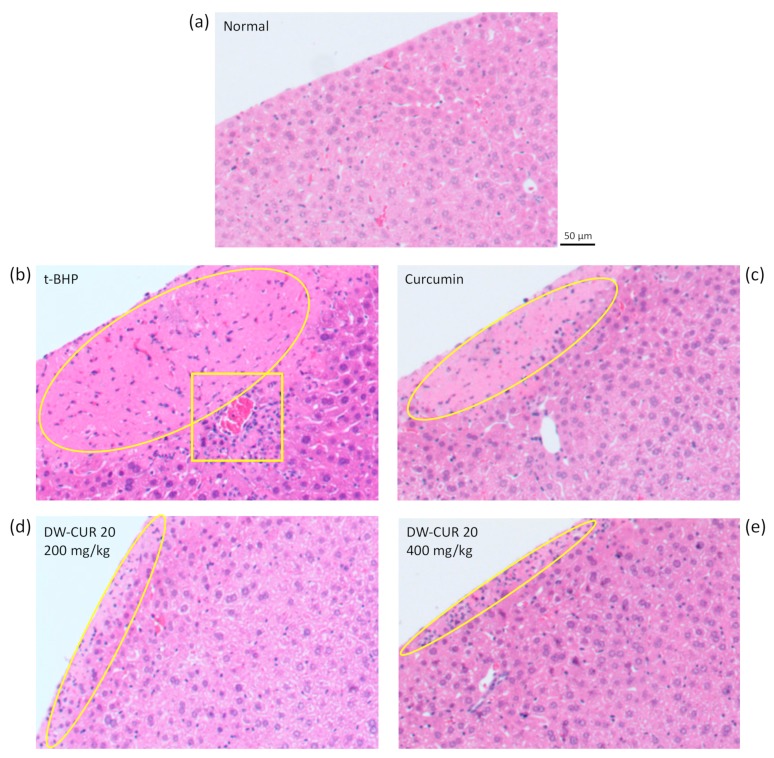
Representative micrograph of hematoxylin and eosin (H&E) stained liver section. (**a**) Liver section of normal mouse. (**b**) Liver section of t-BHP injected mouse showed a wide area of necrotic lesion from the margin of the liver (circle), and excessive cell infiltration and necrosis around the blood vessel (rectangle). (**c**) Liver section of curcumin (80 mg/kg) treated mice showed a reduced necrotic area and cell infiltration. (**d**,**e**) DW-CUR 20 (200 and 400 mg/kg) treatment resulted in limitation of the cell necrosis area only at the margin of the liver.

To evaluate hepatoprotective effects of DW-CUR 20 on liver tissue, we examined histopathology of liver sections from mice with t-BHP-induced hepatotoxicity using hematoxylin and eosin staining. Liver section of normal group showed normal hepatic architecture and clear shape of liver tissue margin (Figure 8a). However, t-BHP injected groups (t-BHP, curcumin, DW-CUR 20) showed necrosis of hepatocyte at margin of the liver tissue. The liver section of the t-BHP group exhibited the widest region of hepatocyte necrosis from the margin and cell infiltration around the blood vessel (Figure 8b). The liver section of the curcumin group showed a slightly reduced necrotic region and notably decreased cell infiltration (Figure 8c). In particular, the hepatic necrosis and cell infiltration were significantly decreased and disappeared in the liver section of DW-CUR 20 (200 and 400 mg/kg) groups.

## 4. Discussion

To date, there have been many attempts to improve the bioavailability of curcumin. These studies improved its bioavailability by adopting various pharmaceutical formulations such as nanoparticles, liposomes, adjuvants, micelles, the phospholipid complex and phytosomes [32]. Table 2 shows the C_max_ and AUC values of curcumin for the representative curcumin formulations reported previously. Most of the formulations exhibited 2–10-fold increases in oral AUC compared with non-formulated curcumin in rats. DW-CUR 20 generally resulted in greater efficiency than the other formulations, except for one formulation based on a solid lipid nanoparticle that resulted in a 39-fold increase in AUC.

Recently, Fan et al. [33] reported that HPMC is useful as an auxiliary excipient in maintaining stability and improving the solubilization ability during the preparation of Eudragit E100-based amorphous solid dispersions of curcumin. According to their report, the dissolution rates, stability, and solubilizing ability of the curcumin/Eudragit E100 formulation were significantly increased by the addition of HPMC. Another report by Fan et al. [34] demonstrated that HPMC interacts with curcumin to generate amorphous solid dispersions through hydrogen bond interactions, which contribute to inhibition of crystallization and enhancement of drug membrane permeability. However, improvements of curcumin bioavailability in the amorphous solid dispersions with HPMC were not investigated in those studies.

The hepatoprotective effect of curcumin has been broadly studied by various research groups, but, to the best of our knowledge, there has been no study to date using t-BHP-induced hepatotoxic model. An organic peroxide t-BHP is metabolized by glutathione peroxidase and cytochrome P450 in hepatocytes which in turn depletes glutathione and initiates lipid peroxidation, and affects the integrity of cellular membrane resulting in cell death [35]. Therefore, t-BHP has been used as a model substance for the evaluation of the pathogenetic mechanisms involved in toxic liver injury and nonalcoholic fatty liver disease due to oxidative stress in cells as well as in animals [36,37,38,39]. In this study, DW-CUR 20 showed protective effects against t-BHP-induced hepatotoxicity in HepG2 cells. Its protective effects were better than the equivalent concentration of pure curcumin in the apoptosis-related protein inhibition although in cell viability and LDH release assays, and the protective effects of DW-CUR 20 was similar to those of curcumin.

It has been generally recognized that a serious limitation for the application of curcumin is its very low oral bioavailability. However, as shown in Table 1, DW-CUR 20 has improved the oral bioavailability and overcome the shortcoming of curcumin. As the bioavailability of oral formulations largely affects it therapeutic efficacy, it was expected that the hepatoprotective effect of DW-CUR 20 in vivo would be potentiated compared with pure curcumin. The present results demonstrated the enhanced hepatoprotective effects of DW-CUR 20 in the mice model of t-BHP-induced acute liver injury. Oral administration of DW-CUR 20 resulted in decreases of serum ALT and AST levels and amelioration of liver histological damage. Moreover, the effects at 200 mg/kg of DW-CUR 20 (equivalent to curcumin 40 mg/kg) were even better than those at 80 mg/kg of curcumin. These increased hepatoprotective efficacy of DW-CUR 20 in mice can be explained in terms of greatly enhanced bioavailability of curcumin.

## 5. Conclusions

Our study demonstrated that the HPMC-based amorphous solid dispersions of curcumin exhibited significantly enhanced oral bioavailability in rats. In addition, we presented its hepatoprotective effects along with the relevant molecular mechanisms. The present results demonstrated that DW-CUR 20 acts as a potential protective agent against t-BHP-induced cell death and hepatotoxicity in mice. This finding thus provides another insight into understanding the underlying mechanisms of the hepatoprotective effect of curcumin. In addition, this study affirms the potential role of curcumin as an antioxidant agent for the prevention of oxidative stress-related liver injury. When considering the efficiency, safety, and simplicity of the preparation process, DW-CUR 20 is a promising formulation for enhancing the therapeutic effects of curcumin and improving its safety.

## Figures and Tables

**Table 1:** Pharmacokinetic parameters for curcumin and DW-CUR 20.

	Parameter	Value
Curcumin	C_max_ (ng/mL)	65.8 ± 21.5
	T_max_ (h)	7.54 ± 9.07
	AUC_last_ (ng⋅h/mL)	781.8 ± 256.5
DW-CUR 20	C_max_ (ng/mL)	4135.0 ± 808.9 ***
	T_max_ (h)	1.6 ± 0.55
	AUC_last_ (ng⋅h/mL)	13,528.4 ± 3025.8 ***

*** *p* < 0.001 compared to “Curcumin” group.

**Table 2:** The peak plasma concentration (Cmax) and the area under the plasma concentration-time curve (AUC) values of curcumin for different curcumin formulations.

Formulations	Dose (as Curcumin)	Cmax (ng/mL)	AUC (ng·h/mL)	Fold Change in AUC	Ref.
Curcumin with tween 80	50 mg/kg	292 ± 100	1075 ± 120	39	[25]
Curcumin solid lipid nanoparticle	50 mg/kg	14290 ± 4290	41990 ± 6180		
Curcumin dispersion	100 mg/kg	120	2588	1.6	[26]
Curcumin nanoemulsion	100 mg/kg	140	4113		
Curcumin suspension with 4% CMC	50 mg/kg	2120 ± 340	10140 ± 610	12.3	[27]
Curcumin solid lipid nanoparticle	50 mg/kg	7510 ± 440	124510 ± 14530		
Free curcumin	10 mg/kg	704.68 ± 73.18	2181.91 ± 195.04	2.0	[28]
Curcumin-chitosan-pectinate-nanoparticle	10 mg/kg	1000.57 ± 15.23	4479.50 ± 137.00		
Curcumin with 0.5 % CMC	50 mg/kg	5.08 ± 1.18	27.45 ± 8.09	3.7	[29]
Curcumin nanosuspension (Brij78)	50 mg/kg	110.01 ± 30.71	101.59 ± 35.29		
Curcumin	100 mg/kg	266.7	2609.04	3.3	[30]
Phospholipid complex	100 mg/kg	600.93	8772.57		
Curcumin suspension (non-formulated)	250 mg/kg	90.3 ± 15.5	312 ± 43	10.3	[31]
Curcumin nanoparticle (w/piperine)	100 mg/kg	260.5 ± 26.4	3224 ± 329		
Curcumin in water (non-formulated)	50 mg/kg	65.8 ± 21.5	781.8 ± 256.5	17.3	This study
DW-CUR 20	50 mg/kg	4135.0 ± 808.9	13,528.4 ± 3025.8		
